# Reliability of an Integrated Ultrasound and Stereophotogrammetric System for Lower Limb Anatomical Characterisation

**DOI:** 10.1155/2017/4370649

**Published:** 2017-06-19

**Authors:** Frederick Greatrex, Erica Montefiori, Thomas Grupp, Josef Kozak, Claudia Mazzà

**Affiliations:** ^1^Department of Mechanical Engineering and INSIGNEO Institute for in silico Medicine, University of Sheffield, Sheffield, UK; ^2^Research & Development, Aesculap AG, Tuttlingen, Germany; ^3^Department of Orthopaedic Surgery, Physical Medicine & Rehabilitation, Campus Grosshadern, Ludwig Maximilians University Munich, Munich, Germany

## Abstract

**Background:**

Lower extremity analysis for preoperative total knee and hip arthroplasty routines can increase surgery success rate and hence reduce associated costs. Current tools are limited by being invasive, limited to supine analysis, or too expensive. This study aimed to propose and validate a device, OrthoPilot®, based on the combined use of a stereophotogrammetric and ultrasound system which can in vivo and noninvasively measure varus/valgus, flexion/extension, femur and tibia torsion, and femur and tibia lengths.

**Methods:**

A phantom was measured by four operators to determine the resolution of the system. Interoperator variability was measured on three operators who measured the above six variables on both legs of three subjects in standing and supine positions. Intraoperator variability was assessed on data from three repeats from 9 subjects (18 legs).

**Results:**

All 6 variables were reliably detected on a phantom, with a resolution of 1 mm and 0.5°. Inter- and intraoperator consistency was observed for varus/valgus, flexion/extension, and length measurements on the healthy subjects in standing and supine positions (all ICC > 0.93). For torsion measurements, there was a considerable variation.

**Conclusion:**

The proposed system, when used on healthy subjects, allowed reliable measurements of key parameters for preoperative procedures in both supine and standing positions. Accuracy testing and further validation on patient populations will be the next step toward its clinical adoption.

## 1. Introduction

Currently, 6–12% of total knee and hip arthroplasties require revision after 10 years [1]. Alongside the fact that there is a predicted 700% rise in total knee arthroplasty (TKA) by 2030 in the USA alone means that such revision rates (including the revision rate which increases beyond the 10 year mark) will equate to a very large number of people and significant cost [2]. Preoperative, lower extremity kinematic evaluations for total hip and knee arthroplasties are therefore considered crucial for successful surgery and patient satisfaction [3, 4]. Accurate determination of kinematic parameters such as the varus/valgus is a key factor for correct surgical planning [5]. Typically, preoperative procedures consist of invasive measures such as CT scans or standing radiographs [6, 7]. Albeit the clinical gold standards, these methods have conveyed unreliability in their analysis. For example, radiographs are subject to magnification effects and errors associated with limb rotation [8–10]. Their poor correlation with navigated measurements taken intraoperatively also raises doubt in their reliability [11]. For repeated measurements, accumulative radiation dosage is also an issue, and as such, less or noninvasive analysis becomes desirable. EOS systems have shown significant radiation reduction and similarly reliable results in their analysis [12, 13]. EOS also has the ability to analyse subjects in various positions [12]. However, its current availability for clinical settings is limited due to its market access [14]. Other analysis has shown to be promising in preoperative routines, be it standing MRI techniques or gait analysis which noninvasively assesses lower extremity kinematics [15–18]. Easily obtaining information between weight-bearing and nonweight-bearing positions could be desirable but currently relies on multiple imaging techniques and has not been extensively researched [5, 19, 20]. This flexibility is not achievable with current gold standard techniques. Useful information is hence potentially overlooked in surgical planning.

Previous studies have successfully measured leg length discrepancy and femur torsion values with ultrasound in the past [21–23]. Studies which integrate motion capture have investigated lower extremity parameters such as hip joint centre calculations and torsion measurements, but not extensive analysis of lower limb alignments [24–26]. Despite being very encouraging for further exploitation of these techniques, none of these studies provided a thorough analysis of all the variables that might be needed for fully determining lower limb alignments for a preoperative analysis.

The OrthoPilot (Aesculap AG, Tuttlingen) system is a medical navigation system most commonly known for its use in computer-assisted surgery in knee, hip, and spine procedures. During surgery, it relies upon registration processes which aid the surgeon in identifying landmarks necessary for optimising the surgical outcome [27]. The aim of this study was to validate the use of OrthoPilot in its integration with an ultrasound system for pre- and postoperative noninvasive lower limb kinematic analysis, by assessing its accuracy and reliability in determining lower limb alignments in standing and supine positions.

## 2. Materials and Methods

**Figure 1:**
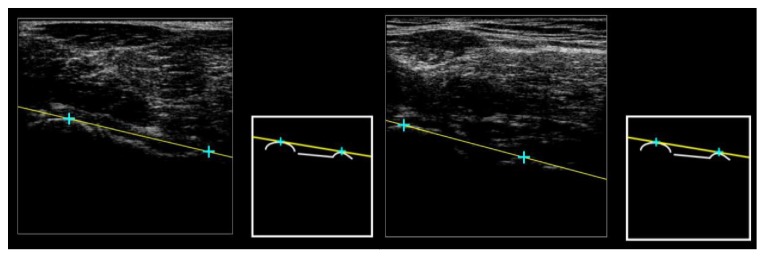
Ultrasound images of the femoral neck for subject S001 (left) and subject S002 (right) with the two points palpated in the immediate postprocessing to determine the femur neck axis. All nine landmarks which were found by the operator are guided by a pattern which is displayed to the right of the ultrasound feed. The guide is shown without the lines/points during the measurement process and with the lines/points in the immediate postprocessing (shown above in both images).

The OrthoPilot comprises two infrared cameras (NDI Polaris Spectra optical tracking system), to be used in combination with two, precalibrated marker clusters. One is attached with a Velcro strap on the proximal tibia, and the other is attached to the ultrasound probe. The ultrasound device (transducer with a frequency of 7 MHz with 128 piezoelectric elements and a width of 90 mm has a penetration depth of 60 mm, an axial resolution of 0.3 mm and a lateral resolution of 0.5 mm) is integrated into the system, and synchronised software is used to measure six variables of interest (varus/valgus, flexion/extension, femur and tibia lengths, and femur and tibia torsions) from the acquisition of a series of ultrasound images (see example in Figure 1) and the following calculations:

- Two images determine the hip joint centre (HJC), one transverse and one longitudinal. In the immediate postprocessing step, circles are then manually fitted to the femoral head curvature and cross-correlated to determine the HJC.
- Two images are needed to calculate femoral torsion, a transverse image along the axis of the femoral neck (Figure 1) and a transverse image of the posterior femoral condyles. The relative difference in the angle formed by the two lines in the transverse plane, manually fitted during the postprocessing, provides the final angle.
- Three images are needed for the tibia torsion, one transverse, posterior image of the proximal tibia plateau, a transverse image of the anterior distal tibia, and a transverse image of the talus. The latter two images form the ankle joint axis, and the relative difference in the angle formed by the ankle joint axis and tibia plateau in the transverse plane, fitted during the postprocessing, represents the torsion angle.
- For the calculation of the knee joint centre (KJC), one image is captured of the femoral trochlea notch. A single point is palpated on the image in the postprocessing. The distance from the HJC to this point calculates the femur length.
- The ankle joint centre (AJC) is calculated by two images. A transverse image of the talus to calculate its midpoint and orientation. The second image is a longitudinal capture of the tibia-talus interface. A single point is then palpated to estimate the depth of the AJC which is then cross-correlated with the midpoint of the talus to calculate the AJC.
- The tibia length is calculated from the KJC to the AJC.
- Varus/valgus is calculated as the relative angle between the femur and tibia length vectors in the frontal plane, also described as the hip-knee-ankle angle (HKA) [5, 28–30].
- Flexion/extension is calculated as the relative angle between the femur and tibia length vectors in the sagittal plane.

For the generation of the parameters in 3D space, the 2D image coordinates of the ultrasound image are converted into the 3D coordinate system of the ultrasound probe's rigid body cluster. Therefore, each pixel on the ultrasound image is displayed in the camera coordinate system. Once the landmark is identified on the ultrasound image during the acquisition, the operator uses a foot pedal to capture the pose of the two rigid bodies which is then temporarily stored on the device. At this point, the 3D coordinates of all image points in the reference (tibia cluster) coordinate system is calculated. This allows small movements of the subject without compromising the calculations, as long as the tibia cluster is not adjusted. This registration process is fully described in [31].

### 2.1. Tests on a Phantom

**Figure 2:**
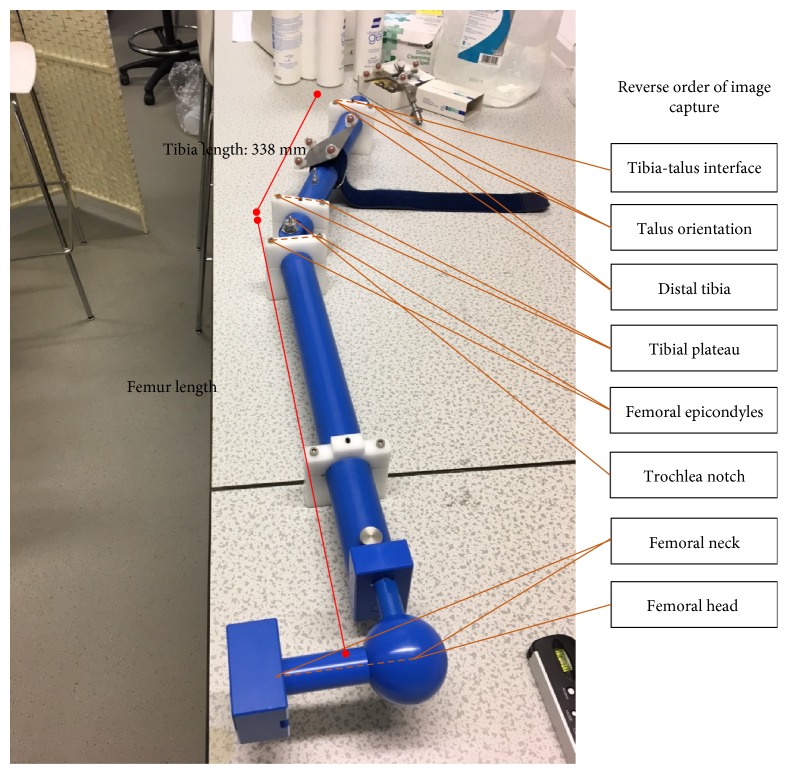
Landmarks needed for full analysis of the phantom, shown at 23° varus for a “left” leg.

Inter- and intraoperator tests with the system have taken place with measurements conducted on a phantom shown in Figure 2. This was to gauge the resolution of the NDI and ultrasound system and assess the repeatability of the measurements in the absence of movement or soft tissue artefacts.

The gold standard used as a reference for the measurements was an inclinometer (Digi-Pas® DWL-80E, SD ± 0.1°), which measured the angles at which the phantom was manually set. Four operators were asked to take complete measurements of the phantom at −15°, −10°, −5°, 0°, 5°, 10°, and 15° (−ve valgus, +ve varus) for a mimicked left leg. An experienced operator conducted three repeats. On the final repeat for each angle, the operator evaluated the same set of images three times in the postprocessing step, outputting five data sets for each angle. This quantified two elements of variability in the testing with OrthoPilot; the identification of the landmarks on the phantom and the immediate postprocessing geometric evaluation of the ultrasound images. Three additional operators conducted one complete measurement of the phantom at the seven chosen angles.

### 2.2. Tests on Healthy Subjects

This study was approved by the University of Sheffield Ethics committee. For the interoperator analysis, the reliability of the measurements was assessed having three operators (OP001, OP002, and OP003) repeating all the measurements three times on three young (age 27.7 ± 1.5) healthy male subjects (S001, S002, and S003) of different body sizes (BMI: 19.9 kg/m^2^, 29.9 kg/m^2^, and 26.2 kg/m^2^, resp.). The experiments were repeated on both legs in standing and supine positions. Supine examination took place across a bed and foot rest, with the posterior knee exposed. Standing examination was conducted asking the subjects to keep their feet just over shoulder width apart. All image captures were performed in the same order for standing and supine analysis, as previously described, on both legs. A power analysis based on the data from the interoperator analysis showed that to achieve a power greater than 0.8 with a SD of 1°, a sample size of at least 6 subjects was needed for the intraoperator reliability analysis. To this purpose, OP001 conducted the same experiments on six further young healthy subjects (4 males, 2 females, age 27.5 ± 4.5 y.o., and BMI 21.4 ± 4.0 kg/m^2^).

### 2.3. Statistical Analysis

Statistical analysis was performed using SPSS (IBM Corp., Armonk, New York). For phantom measurements, inter- and intraoperator analysis was performed by assessing the mean and standard deviation (SD) of all variables at each angle. Bland-Altman analysis was performed to interpret the level of agreement between the true and measured value of varus/valgus. Comparisons were initially tested for normality (Shapiro-Wilk test).

For analysis on healthy subjects, the mean and SD were calculated in both supine and standing positions. Interoperator reliability was assessed using a two-way random effects model. The intraclass correlation coefficient (ICC) with 95% confidence intervals (CI) was computed across the three operators for all the variables in the standing and supine positions. For this analysis, subject legs were not considered individually. To complement these results, a randomised one-way analysis of variance (ANOVA) was used to determine whether there were significant differences between the mean standard deviations of the three operators (alpha level set at 0.05 for all tests).

## 3. Results

### 3.1. Phantom Tests

The OrthoPilot provided highly repeatable measurements when used on a phantom (Figure 2) with small measured differences between the operators. The interoperator study showed that varus/valgus measurements were slightly over estimated, with a mean error of 0.4° ± 0.3°. Flexion/extension (set at 0°) and the remaining variables were slightly under estimated (−0.5° ± 0.1°). Femur and tibia torsion values (set at 37° and 89°, resp.) were consistent across all operators (34.8° ± 0.5° and 87.3° ± 0.6°, resp.). Femur and tibia measurements (actual lengths 510 mm and 338 mm, resp.) were consistent for all operators, with a slightly higher range for the femur lengths over the 7 measurements of the phantom (506 mm ± 1.1 mm and 337 mm ± 0.7 mm, resp.).

Intraoperator varus/valgus measurements were also slightly over estimated, with a mean error 0.3° ± 0.2°. Flexion/extension was underestimated (−0.7° ± 0.3°) and similarly, the remaining variables were also underestimated. Femur and tibia torsion values were 35.0° ± 0.5° and 86.0° ± 0.7°, respectively. Femur and tibia lengths showed consistency and were 508 mm ± 1.4 mm and 335 mm ± 0.6 mm, respectively.

For the variability in geometric evaluation, the highest error, albeit small, was shown for the segment length calculations with a standard deviation of ±0.6 mm occurring in six out of the fourteen measurements (femur and tibia). In the remainder, it was 0 mm. For the varus/valgus, flexion/extension, femur and tibia torsion angles, the largest SD was ±0.2°, with an average of ±0.1°.

**Figure 3:**
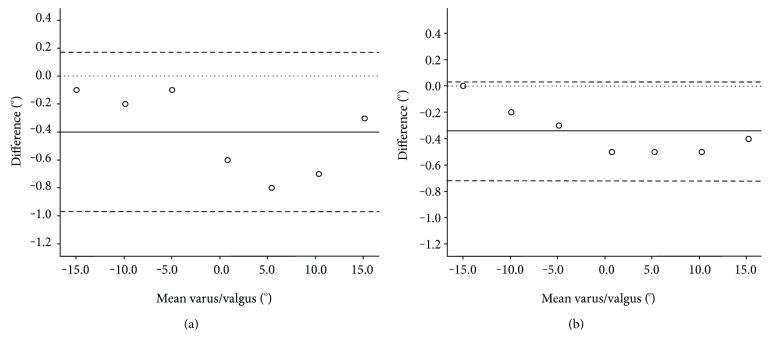
Inter- (a) and intraoperator (b) Bland-Altman plots of the difference between the measured and actual varus/valgus angle (°) plotted against the mean of the measured and actual angle. The mean (solid line), zero (dotted line), and limits of agreement at ±1.96 SD (dashed lines) are shown.

Figure 3 shows Bland-Altman plots for the varus/valgus measurements and visually interprets the comparison in bias between inter- and intraoperator results. A slightly better agreement is observed for the intraoperator results (−0.7°–0.0° compared to −1.0°–0.2°).

### 3.2. Tests on Healthy Subjects

Data was recorded without particular difficulties by the three operators. For the measurement of one limb, the whole process took on average 8 minutes with 2-3 minutes spent on ultrasound image capturing and 1-2 minutes on the immediate postprocessing. In the standing position, the image capturing process was slightly longer, with an average time of 10 minutes for the whole process.

All interoperator values are reported in Table 1, which shows the means and SD for each operator for all variables in standing and supine. For all measures of length, in standing and supine, ICC values were >0.99. For measures with reference to these length vectors (varus/valgus and flexion/extension), in standing and supine, ICC values were >0.93. For measures of the torsions, except supine femur torsion measurements, the ICC drops to lower than 0.70 for the tibia torsion. For the ANOVA, all tests showed no significance between the standard deviations of each operator for all variables in supine and standing (*p* > 0.17). For the intraoperator results, varus/valgus and flexion/extension measurements were an average of 1° of SD across nine subjects (18 limbs) for OP001. The same results were observed also for OP002 and OP003, when looking at corresponding data from three subjects (6 limbs). The length measurements were also very consistent with few SD exceeding 5 mm. For both femur and tibia torsions as measured by OP001 across nine subjects, the average SD was less than 4°. Many measurements, however, exceeded 5° of SD for OP002 and OP003.

## 4. Discussion

TKA preoperative analysis of key lower extremity variables such as varus/valgus and femur torsion is vitally important for enhanced surgical planning [3, 4]. This study aimed to validate a device which could potentially eradicate the use of invasive methods, such as full-length X-rays, with the use of an ultrasound-integrated motion capture system. In addition, its suitability to measure the anatomical variables of interest for surgical planning in standing (weight bearing) and supine (surgery-like) positions was shown.

Reported results showed that the OrthoPilot can detect both joint angles and segment lengths when used on a phantom to within a reliable resolution. The results are comparable with the length measurements performed by Escott et al. [13], who compared four different measurement techniques (standard radiographs, CT scans, EOS-slow, and EOS-fast) for leg length measurements with respect to a phantom of known length. EOS-slow measurements performed most accurately with an average 0.5% underestimation of the phantom length. In this study, the smallest and largest underestimation from the true value for the tibia and femur phantom measurements was 0.3% and 0.8% of their length, respectively. A key limitation of this part of the study, aiming at testing the resolution of the measurement devices, was that actual accuracy of the system was not quantified either on more realistic phantoms (e.g., adding silicon pads with plastic bones in water) or even better using alternative imaging systems (e.g., CT scans) on the healthy subjects. Further studies will focus on these aspects.

For lower limb segment length measurements on subject cohorts, few studies have been conducted using ultrasound [21, 32]. Most studies use conventional means such as radiographs or MRI [13, 33–35]. Terjesen et al., however, showed that 95% of the ultrasound leg length measurements on 45 subjects were within 7 mm of the radiographic measurements [21]. Their follow-up study [32] further emphasised the potential for ultrasound as a clinical tool with a study on 100 healthy subjects and showed that 95% of the differences for the length measurements between the two operators were less than 5 mm. Similar repeatability values were found in this study, the average SD for both femur and tibia lengths over three repeats for all operators on both limbs was 3 mm ± 2 mm (with highest value being 11 mm).

For varus/valgus measurements on subject cohorts, standing radiographs, CT scans, or MRIs are the most common choice for analysis, depending on availability. On patients, studies have taken place to determine whether such methods correlate with measurements taken intraoperatively, whereby surgeons have attempted to measure varus/valgus with a navigation system before proceeding with the surgery [11]. Yaffe et al. showed a large discrepancy between preoperative standing radiographs and preoperative navigation measurements (4.7° ± 2.9° difference) [11]. In this case, however, it was noted that the difference may have resulted from weight-bearing against nonweight-bearing analysis and its influence on lower limb kinematics which has shown to be significant [5, 18]. Bellemans et al. investigated the HKA angle with full weight-bearing radiographs on 250 males and 250 females, a considerably larger cohort than this study. Their varus/valgus measurements performed on young, healthy subjects suggested a certain degree of varus deformation is more likely than neutral alignment (average of 1.9° in males and 0.8° in females) [36], which is consistent with the values found in this study (average of 0.2° and 0.3° in standing and supine, resp.).

Although the knee flexion/extension angle may not immediately seem like an important parameter in the context of surgical planning, it has actually been shown that it affects the varus/valgus when analysed on cadavers in the frontal plane [27]. This might have indeed affected some of the results of this study, where a larger knee flexion of ≈15° was observed, on average, by all operators for subject S001 in the standing position. Whilst this angle was surprisingly large, it was double checked with a goniometer, which provided consistent findings. Hauschild et al. showed that with increasing amounts of flexion, the reliability of the measurements decreases significantly compared to extended measurements. Therefore, controlling for the flexion/extension when measuring the varus/valgus is important. Further studies will investigate this observation more thoroughly in a patient cohort.

Kulig et al. validated an ultrasound graphic-tilting method based on the use of an inclinometer attached to an ultrasound probe. This technique highly correlates with MRI data for measurements of the femur torsion [23]. Hudson et al. used a similar technique and found much higher interoperator reliability for the tibia torsion measurements (ICC > 0.84) than those found in this study [22]. Both studies showed average tibia torsions which were comparable to our findings but their femur torsion measurements are considerably lower. The CI values in this study, conversely, portrayed a lack of interoperator reliability. The negative CI values suggest an intraoperator variability which exceeds the interoperator variability. A reason for this inconsistency, especially for the tibia torsion, is likely due to the measurements needed for its calculation. Three images are needed, the tibia plateau and two for the ankle joint axis. This dependence on three images compared to two for the femur torsion potentially increased the variability between the operators. Further studies are needed to fully elucidate this aspect.

The above results are likely linked to one of the main limitations encountered in this study, which was the difficulty in the ultrasound image capturing associated with the application of pressure along the entire surface of the probe, especially in the standing position. For subjects which have low BMI (S001), this is not much of a concern. However, as tissue artefacts increase, optimal ultrasound image generation might become a problem, possibly causing restrictions in the systems' capability for high-BMI individuals, as also suggested by the error for the femur torsion measurements in S002. Imaging the femur neck orientation, for example, required an equal pressure distribution over a relatively large probe face (90 mm length). This point ties in with operator experience and possible varying interpretations of the ultrasound images [37]. OP003 had been properly trained and went through various sessions to familiarise themselves with the procedures, but their knowledge and experience with ultrasound were lower than the other operators. Also, all the images were captured without any adjustment of the ultrasound parameters between trials and operators. Altering the ultrasound settings and using a smaller probe in the standing position might have led to even better results. It must also be emphasised, however, that there was difficulty in measuring some of the points in the standing position and this was potentially pushing the system beyond what it was originally designed for. For example, the measurement of the femur trochlea notch in the standing position was the most problematic point and is a single measurement which influences both the femur length and the varus/valgus values.

The subject cohort size for this study was small, which of course does not allow for generalisation about the changes observed between the supine and standing positions. Conclusions are hence limited to the reliability and feasibility of the proposed approach. According to the reported results, especially for length, varus/valgus and flexion/extension measurements, these can be deemed satisfactory.

## 5. Conclusion

With the correct expertise and knowledge of ultrasound, the system portrayed in this study is easy and efficient to use. With simple and integrated image postprocessing, the system determines key lower limb kinematic parameters reliably on both a phantom and healthy subjects. Alterations in the flexibility of the ultrasound device will be investigated as this could prove useful in high-BMI subjects. In a clinical setting, the ability to take measurements in supine and standing positions with one measurement device could possibly enhance surgical planning. The noninvasive nature and speed of the system compared to alternatives in lower limb analysis are coherent reasons to warrant further investigation.

## Figures and Tables

**Table 1:** Individual and overall results for the three operators across *n* subjects (2*n* limbs) for all 6 variables in standing and supine positions.

Variable	Position	OP001 (*n* = 9)	OP002 (*n* = 3)	OP003 (*n* = 3)	Average^¥^ (*n* = 3)	ICC (95% CI) ^§^ (*n* = 3)	*p* value^¢^
Varus (+ve)/ valgus (−ve) (°)	Standing	0.2 ± 3.2	0.1 ± 2.6	0.3 ± 3.2	0.2 ± 2.8	0.97 (0.86–0.99)	0.61
	Supine	1.1 ± 2.7	0.4 ± 1.8	0.0 ± 1.6	0.3 ± 1.8	0.93 (0.71–0.99)	0.63
Flexion (+ve)/ extension (−ve) (°)	Standing	0.7 ± 6.2	4.2 ± 8.4	3.9 ± 8.5	3.6 ± 8.1	0.99 (0.97–0.99)	0.39
	Supine	1.3 ± 3.4	1.8 ± 4.5	2.0 ± 4.0	1.7 ± 4.2	0.99 (0.97–0.99)	0.80
Femur length (mm)	Standing	435 ± 31	457 ± 37	457 ± 35	456 ± 35	0.99 (0.99–1.00)	0.66
	Supine	439 ± 29	454 ± 34	456 ± 33	455 ± 33	0.99 (0.99–1.00)	0.49
Tibia length (mm)	Standing	410 ± 27	426 ± 22	429 ± 23	428 ± 22	0.99 (0.98–0.99)	0.55
	Supine	406 ± 25	427 ± 21	424 ± 22	425 ± 21	0.99 (0.98–0.99)	0.17
Femur torsion (°)	Standing	32.7 ± 10.2	27.2 ± 16.0	36.5 ± 10.3	31.7 ± 12.2	0.68 (−0.09–0.95)	0.43
	Supine	28.6 ± 9.6	25.0 ± 17.9	26.2 ± 12.0	26.2 ± 13.3	0.95 (0.78–0.99)	0.74
Tibia torsion (°)	Standing	30.9 ± 9.4	28.0 ± 6.9	28.2 ± 12.5	28.5 ± 10.4	0.69 (−0.61–0.96)	0.17
	Supine	32.1 ± 8.5	32.4 ± 7.8	36.6 ± 8.6	33.5 ± 8.9	0.65 (−0.35–0.95)	0.78

Mean and SD of values measured by each operator over *n* subjects. ^¥^Mean and SD across all operators for the same three subjects. ^§^Intraclass correlation coefficient (ICC) and 95% confidence intervals (CI). ^¢^One-way ANOVA values.
